# Residential exposures associated with adiposity and proinflammatory markers in obese adults with metabolic syndrome: a cross-sectional study

**DOI:** 10.2478/aiht-2026-77-4124

**Published:** 2026-09-25

**Authors:** Angel M. Dzhambov, Spas Kitov, Kostadin R. Kostadinov, Maria-Florance Kitova, Angel Burov, Marco Helbich, Tanya Deneva, Lyudmila Kitova

**Affiliations:** Medical University of Plovdiv, Strategic Research and Innovation Program for the Development of MU – Plovdiv, Health and Quality of Life in a Green and Sustainable Environment Research Group, Plovdiv, Bulgaria; Medical University of Plovdiv, Research Institute at Medical University of Plovdiv, Environmental Health Division, Plovdiv, Bulgaria; Medical University of Plovdiv, First Department of Internal Diseases, Plovdiv, Bulgaria; St George University Hospital, Clinic of Cardiology Plovdiv,Bulgaria; Medical University of Plovdiv Faculty of Public Health, Department of Social Medicine and Public Health, Plovdiv, Bulgaria; Medical University of Plovdiv Faculty of Medicine, Plovdiv, Bulgaria; University of Architecture, Civil Engineering, and Geodesy, Faculty of Architecture, Department of Urban Planning, Sofia, Bulgaria; Utrecht University, Faculty of Geosciences, Department of Human Geography and Spatial Planning, Utrecht, The Netherlands; Medical University of Plovdiv, Faculty of Medicine, Department of Clinical Laboratory, Plovdiv, Bulgaria

**Keywords:** adipokines, air pollution, greenspace, systemic inflammation, traffic noise, adipokini, onečišćenje zraka, prometna buka, sistemska upala, stambeno zelenilo

## Abstract

Although current research supports associations between residential environment and inflammation and/or adiposity, evidence remains limited and partly contradictory for combined exposures, especially in vulnerable populations. To address this issue, this cross-sectional study, taking place in 2023 and 2024, investigated the associations between residential factors and adiposity and proinflammatory markers in 90 obese adults with metabolic syndrome from Bulgaria. All underwent clinical, anthropometric, and laboratory examinations, and completed a questionnaire. We measured their serum proinflammatory cytokines [interleukin-6 (IL-6), tumour necrosis factor alpha (TNF-α)], C-reactive protein (CRP), and adipokines (visfatin, leptin, adiponectin). Adiposity-related markers included body mass index (BMI), waist-to-hip ratio (WHR), body fat, and visceral fat percentage. Residential air pollution (NO_2_, PM_2.5_, PM_10_), road traffic noise (L_den_), domestic burning of solid fuel, total greenness, tree cover, and time spent in nature were linked to these data. There was suggestive evidence that every 10 μg/m^3^ increase in NO_2_ levels was associated with higher concentrations of TNF-α (36.27 %; 95 % CI: −5.66 %, 96.83 %), IL-6 (41.63 %; 95 % CI: 8.16 %, 85.46 %), CRP (37.80 %; 95 % CI: 5.89 %, 79.32 %), leptin (25.61 %; 95 % CI: −8.73 %, 72.88 %), and lower adiponectin-leptin ratio (−33.83 %; 95 % CI: −56.59 %, 0.85 %). Additionally, NO_2_ was associated with higher BMI (6.37 %; 95 % CI: −0.82 %, 14.08 %) and visceral fat (7.52 %; 95 % CI: 1.30 %, 14.12 %). For every 2 h/week spent in nature, TNF-α concentration was lower (−20.49 %; 95 % CI: −37.56 %, 1.23 %), and adiponectin was higher (10.30 %; 95 % CI: 1.36 %, 20.03 %). Other observed associations were largely null, inconsistent, or occasionally showed an unexpected protective direction. These findings can serve as a general roadmap, and only larger longitudinal studies may provide a better insight into the environmental determinants of metabolic inflammation, which could in turn support the development of alternative preventive strategies.

## Abstract (hr)

Iako dosadašnja istraživanja upućuju na povezanost stambenog okoliša s upalom i/ili adipoznošću, dokazi o povezanosti kombiniranih izloženosti i dalje su ograničeni i djelomično proturječni, osobito u ranjivih populacija. Ovim presječnim istraživanjem, provedenim 2023. i 2024. godine, ispitane su povezanosti čimbenika stambenog okoliša s adipoznošću i proinflamatornim biljezima u 90 pretilih odraslih osoba s metaboličkim sindromom iz Bugarske. Svi su ispitanici podvrgnuti kliničkim, antropometrijskim i laboratorijskim pretragama te su ispunili upitnik. Izmjerene su koncentracije proinflamatornih citokina u serumu [interleukin-6 (IL-6), čimbenik nekroze tumora alfa (TNF-α)], C-reaktivnoga proteina (CRP) i adipokina (visfatina, leptina, adiponektina). Biljezi povezani s adipoznošću uključivali su indeks tjelesne mase (ITM; eng. BMI), omjer opsega struka i bokova (OSB; eng. WHR), udio tjelesne masti i udio visceralne masti. Za te smo parametre utvrdili određene povezanosti s podacima o onečišćenju zraka u stambenom okolišu (NO_2_, PM_2,5_, PM_10_), buci cestovnoga prometa (L_den_), izgaranju krutih goriva u kućanstvu, ukupnom zelenilu, pokrivenosti krošnjama drveća i vremenu provedenom u prirodi. Postoje naznake da je svako povećanje koncentracije NO_2_ za 10 μg/m³ povezano s višim koncentracijama TNF-α (36,27 %; 95 % CI: −5,66 %, 96,83 %), IL-6 (41,63 %; 95 % CI: 8,16 %, 85,46 %), CRP-a (37,80 %; 95 % CI: 5,89 %, 79,32 %) i leptina (25,61 %; 95 % CI: −8,73 %, 72,88 %) te nižim omjerom adiponektina i leptina (−33,83 %; 95 % CI: −56,59 %, 0,85 %). Osim toga, NO_2_ bio je povezan s višim ITM-om (6,37 %; 95 % CI: −0,82 %, 14,08 %) i većim udjelom visceralne masti (7,52 %; 95 % CI: 1,30 %, 14,12 %). Na svaka dva sata tjedno provedena u prirodi koncentracija TNF-α bila je niža (−20,49 %; 95 % CI: −37,56 %, 1,23 %), a koncentracija adiponektina viša (10,30 %; 95 % CI: 1,36 %, 20,03 %). Ostale utvrđene povezanosti uglavnom nisu bile statistički značajne ili dosljedne ili su povremeno upućivale na neočekivani zaštitni učinak. Ovi rezultati mogu poslužiti kao opći putokaz, a tek veća longitudinalna istraživanja mogla bi pružiti bolji uvid u okolišne odrednice metaboličke upale, što bi zauzvrat moglo pridonijeti razvoju alternativnih preventivnih strategija.

The global burden of obesity and its associated health implications have increased 2.5 times over the last 30 years (1). Besides genetic and lifestyle factors, several environmental factors likely contribute to obesity (2, 3), and there is evidence suggesting that the built environment may have an obesogenic effect (4). For instance, an association has been reported between air pollution and obesity (5), primarily through oxidative stress and inflammation (6). Oxidative stress and inflammation can occur not only in the respiratory system but also move to systemic circulation through the spill-over of inflammatory mediators from the lungs or by ultrafine particles reaching target organs directly (7).

Systemic inflammation is the underlying cause of the development and progression of multiple cardiometabolic, neurological, allergic, and mental health conditions (8). While short-term exposure to proinflammatory cytokines such as interleukin-6 (IL-6) and tumour necrosis factor-α (TNF-α) is associated with immune response activation against infectious agents and malignant cells (9), chronic low-grade inflammation can damage cells and tissues through adverse metabolic, signalling, and epigenetic pathways (10). Research into the role of metabolic inflammation in obesity has revealed that high concentrations of circulating proinflammatory agents in obese individuals further increase the risk of cardiometabolic complications (9). A vicious circle forms with inflammation promoting adipose tissue accumulation through dysregulation of carbohydrate and lipid metabolism and impaired insulin sensitivity, while proinflammatory cytokines and specific adipokines are, in turn, secreted by adipocytes (11).

Both traffic-related (12, 13) and household-related air pollution from burning fossil fuels (14) have been associated with increased circulating cytokines, though the evidence for long-term effects varies by inflammatory biomarkers (12). Fewer studies have considered the effect on adipokines, but some have found that air pollution increases leptin (15), which is associated with inflammatory effects (16), and decreases adiponectin, which is typically associated with reduced cardiometabolic risk (17). However, other proinflammatory adipokines, such as visfatin (18), have not yet been researched in relation to environmental influences.

Compared with air pollution, there has been less research on the inflammatory effects of road traffic noise. More is known of the positive association between exposure to road traffic noise and markers of obesity (19). An analysis of 11 cohorts revealed a higher likelihood of obesity and central obesity with higher road traffic levels (20). Chronic noise exposure contributes to oxidative stress and inflammation (21), as it has been shown to modulate immune function through neuroendocrine pathways and activation of the body's stress axes (22). However, the evidence for these immunological effects originates mostly from experimental animal studies (23) rather than epidemiological studies in humans.

Besides potentially obesogenic and proinflammatory exposures, there are beneficial environmental factors that could act in the opposite direction by supporting a healthier immune response and lower adiposity. For instance, forest bathing (24, 25), more frequent nature contact, spending more time outdoors (26, 27), and living in a greener environment have been associated with anti-inflammatory effects (26, 28). Such benefits can be explained by greenspace, such as parks and natural areas, competing for land use with traffic, promoting outdoor physical activity, and lowering psycho-physiological stress (29). Consequently, activation of these pathways can build stronger biopsychosocial resilience to harmful influences (30).

Although current research indicates that residential environment is related to inflammation and adiposity, evidence remains limited and partly contradictory for combinations of multiple exposures and biomarkers, especially in vulnerable populations (31). Of particular interest are obese individuals, given the significant role played by the inflammatory response in the pathogenesis of obesity-related chronic diseases (10). For instance, individuals with prediabetes may be more vulnerable to air pollution. In one study, air pollution was associated with higher levels of leptin and C-reactive protein (CRP) in the prediabetic group than in the non-diabetic group (15). Inflammation could also enhance the effect of air pollution on metabolic syndrome (MetS) (32). Still, whether obese individuals would benefit from improvements in environmental conditions remains unclear.

To address this knowledge gap, we assessed the joint associations of residential exposure to air pollutants, road traffic noise, greenspace availability, and nature contact, with proinflammatory and adiposity-related markers in obese adults. We hypothesised that higher levels of air pollution and traffic noise would be associated with higher concentrations of proinflammatory cytokines and adiposity markers and with lower levels of adiponectin. The opposite was hypothesised for higher greenspace availability and time spent in nature.

## MATERIALS AND METHODS

### Study design and sampling

The study was conducted in Bulgaria, a country with the highest air pollution-related mortality (33) and the shortest life expectancy in the European Union (34). We leveraged data from outpatients of the St George University Hospital Clinic of Cardiology in Plovdiv. As the clinical sample was originally collected to explore multiple outcomes (i.e. electrocardiographic, sleep-related, metabolic, and clinical laboratory markers), analytical sample sizes varied across analyses, as reported in previous studies (35, 36). Initially, obese individuals were recruited between September 2023 and September 2024. The inclusion criterion was diagnosed MetS according to the criteria of the International Diabetes Federation (37). The exclusion criteria were namely chronic renal, hepatic, respiratory, cardiac, and thyroid diseases, cancer, pregnancy, electrolyte imbalance, recent infections, and taking medications known to affect electrocardiographic repolarisation.

For the current secondary analysis, 90 participants, who had previously undergone clinical, anthropometric, and laboratory examinations, gave informed consent to participation. From August to October 2024, they were invited to complete an additional survey concerning their psychological well-being, sociodemographics, lifestyle habits, and residential environment. We recorded their residential address to assign to it environmental exposures. All participants had lived at their present address for at least five years. Since some addresses could not be geocoded (n=7), data for some environmental factors are based on the addresses of 83 participants. About half provided an address in the city of Plovdiv, while the rest lived in other settlements. For geospatial analyses we used the QGIS 3.28.2 geographic information system software (38) to link environmental exposure data to participants' residential addresses.

The study was reviewed and approved by the Scientific Ethics Committee at the Medical University of Plovdiv (Protocol No. 9 of 28/11/2024 and Opinion No. P-KHE-22 of 31/12/2024). Prior to enrolment in the follow-up survey on environmental exposures, participants provided an additional written informed consent for their data to be processed for the current study.

### Inflammatory mediators and adipokines

The study's primary outcomes were proinflammatory cytokines (IL-6, TNF-α), high-sensitivity CRP, and adipokines (visfatin, leptin, and adiponectin) measured at the Central Clinic Laboratory of St George University Hospital. Venous blood samples were collected in accordance with standard procedures, and serum from each participant was aliquoted and stored at −20 °C until analysis, with a maximum storage time of two months. Serum CRP levels were measured by immunoturbidimetry using an AU 480 clinical chemistry analyser (Beckman Coulter, Inc., Brea, CA, USA). The serum concentrations of TNF-α, IL-6, leptin (LDN, Germany), visfatin, and high-sensitivity adiponectin were determined using competitive enzyme-linked immunosorbent assays following local validation and manufacturers' instructions (BioVendor, Asheville, NC, USA). The coefficient of variation for both intra-assay and inter-assay precision was <10 %. We also calculated the adiponectin-leptin ratio as a marker of dysfunctional adipose tissue as described elsewhere (39).

### Adiposity markers

Anthropometric measurements included body mass index (BMI), waist-to-hip ratio (WHR), total body fat, and visceral fat. These measurements were taken at the Department of Physiology of the Medical University of Plovdiv. BMI (i.e., weight in kilograms divided by height in meters squared) was calculated from weight and height measured barefoot and in light clothing. WHR was calculated from hip circumference and waist circumference measured at the midway between the last palpable rib and the iliac crest (40).

Body fat percentage (BF%) and visceral fat percentage (VF%) were estimated based on multifrequency bioelectrical impedance analysis using an InBody_270_ analyser (InBody Co., Ltd., Seoul, South Korea). This technique uses varying frequencies of alternating current that are sent through the body. Their passing through different tissues alters the resulting voltage, which is used to predict BF% and VF%. This renders reasonable estimates compared to the reference method, dual-energy X-Ray absorptiometry (41). To control for factors affecting body water and tissue conductivity, participants were instructed to prepare for this assessment by fasting for at least four hours before measurement, emptying their bladder, abstaining from alcohol for at least 24 h, and avoiding physical strain. They were instructed to lie supine for at least five minutes before testing. Then, the electrode sites were cleaned, and the electrodes were placed on the wrists and ankles. Participants were instructed to keep their limbs separated from their torso and from each other.

### Air pollution

Mean annual concentrations of nitrogen dioxide (NO_2_) and of particulate matter with aerodynamic diameter ≤2.5 μm (PM_2.5_) and ≤10 μm (PM_10_) were obtained from air pollutant levels for the year 2019, mapped at a 25 m spatial resolution using geographically weighted land use regression (LUR) models based on measured pollutant concentrations at monitoring stations across Europe, as described by Shen et al. (42). Predictors included road, land use, satellite retrievals, and chemical transport model estimates. The five-fold cross-validation R^2^ values were 0.66 for NO_2_, 0.77 for PM_2.5_, and 0.62 for PM_10_.

To approximate household air pollution, our survey also included a self-reported item (yes/no) indicating whether the participant's household regularly used solid fuel (coal, firewood, briquettes, or pellets) for heating or cooking.

### Traffic noise

Road traffic noise assessments were taken from the noise map of predicted A-weighted day-evening-night sound levels (L_den_) from road traffic based on long-term land-use regression (LUR) model trained on 232 sites as described elsewhere (43). For Plovdiv, it relied on noise measurements from 44 monitoring sites between 2018 and 2022. Noise levels were predicted using the extreme gradient boosting algorithm and transport- and land-use-related predictors, which achieved the highest fit (R^2^=0.68). However, it could only be applied for participants residing in the city of Plovdiv (n=42), as the remaining participants primarily lived in areas not mapped by the mentioned study (43).

### Greenspace

The availability of green vegetation was defined as the mean normalised difference vegetation index (NDVI) and the percentage of tree cover in a 300 m Euclidean buffer around participants' home addresses as recommended by the World Health Organization (44). NDVI was derived from the local Sentinel-2 radar imagery with cloud cover of less than 10 %, which is part of the Copernicus Programme. NDVI ranges from −1.0 to 1.0, with higher values indicating greater photosynthetically active vegetation biomass (45). The images were captured between May and September 2022, when vegetation is the greenest, and NDVI was calculated at 10 m resolution. Poor-quality surface reflectance values were filtered out using a pixel-based quality check to cloud mask and quality assessment band (QA60) information (46) prior to computing the median NDVI pixel values across overlapping scenes for each address, which was utilised for subsequent analyses.

Pre-processed data on tree cover density were acquired from the 2018 European Urban Atlas with a 10 m resolution, which is part of the Copernicus Land Monitoring Service.

Due to a potentially stronger correlation between actual use of greenspace and health benefits (47), participants were also asked to self-report the amount of time they spent in green spaces or natural areas over a typical week in the last month. Based on the reported visitation frequency and duration, the total time (hours) spent in nature per week was calculated.

### Confounders

To reduce confounding bias, we controlled for both individual and area-level variables. The confounder selection was guided by a directed acyclic graph. Participants reported their age (in years), sex (male/female), self-identified ethnicity (Bulgarian/other), and the highest level of completed education (secondary/higher). The history of smoking was dichotomised as smokers or non-smokers. Self-rated general health was measured on a scale from 1 (very poor) to 5 (very good). Income adequacy represented the self-perceived ability of the household to meet financial demands given its total income and was rated on a scale from 1 (very difficult) to 6 (very easy). The building type was also used as a measure of socioeconomic status, with categories including apartment building, detached house, multi-family house, or other type. City-specific effects were captured using a dummy variable. With half of the participants residing in Plovdiv, this variable was dichotomised as either Plovdiv or another settlement. To capture urbanicity levels within Plovdiv, we computed population density within 300 m buffers around the participants' home address. Population data were obtained from the 2021 census (48).

### Statistical analysis

The data were screened for missing values. The self-reported variable with the largest percentage of missing values (n=25) was perceived income adequacy. Traffic noise data were only available for residences in the city of Plovdiv, and noise levels for all other participants were considered missing. The distributions of IL-6, TNF-α, adiponectin-leptin ratio, and BMI were influenced by a few extreme positive values (49), and these outliers were recoded to just above the highest value not considered an outlier as recommended by Tabachnick and Fidell (50). Rather than conducting complete-case analyses with our limited sample, missing values were imputed for the main multivariate models through multiple imputation by chained equations. Other sociodemographic variables, self-rated health, solid fuel use, city, and building type were used as predictors in the imputation procedure to generate 10 imputed datasets. Kendall's Tau correlations were used to examine bivariate associations between the exposure and outcome variables and potential confounders.

Multivariate regression models were developed to investigate the associations between each outcome and the exposures, including potential confounders. Exposure-outcome associations from the main models were first inspected for nonlinearity using generalised additive models (51, 52). Comparison of alternative degrees of freedom suggested using three degrees of freedom for the estimated smoother. Nonlinearity was inferred based on visual inspection of the smoother and on the significant Gain statistic using the generalised additive model (“gam”) and “gamplot” statistical packages (Stata/MP, version 18, StataCorp LLC, College Station, TX, USA) (53). These tests revealed only a few nonlinear associations, particularly for L_den_ – CRP and L_den_ – BMI and for NDVI – WHR. Therefore, L_den_ was not included in the main models as a categorical variable to prevent further loss of statistical power.

Both single- and multi-exposure regression models were fitted. To address the skewed distributions of the outcome variables, we fitted generalised linear models with gamma distributions and log links, which yielded lower AIC and BIC values and thus a better fit compared with alternative models. To make inferences about the arithmetic mean on the original scale, this approach was preferred over transforming the variables into their natural log and modelling them with an ordinary least squares regression. Robust standard errors were employed to control heteroscedasticity for all regression coefficients. Effect estimates were presented as percentage change in the outcome per unit increase in the respective exposure (i.e. 10 μg/m^3^ in air pollutants, 5 dB in L_den_, 0.1 units in NDVI, 10 % in tree cover, and 2 hrs/week in nature) or for presence v absence of solid fuel burning in the home.

The main models were adjusted for age, sex, ethnicity, education, perceived income adequacy, and city. The models for L_den_, restricted to participants residing in Plovdiv, were additionally adjusted for population in the 300 m buffer instead of the city. To assess the robustness of our findings, we rendered the main models with additional adjustments for self-rated health, building type, and smoking. Multicollinearity tests did not reveal strong correlations among the independent variables (i.e., variance inflation factor <5 and tolerance >0.2). In addition, the main models were rendered for the subgroup of participants from Plovdiv, as this subgroup exhibited greater homogeneity and a divergent pattern of exposure levels than the participants from other settlements. Results were considered statistically significant at p<0.05 (two-tailed). Statistical analyses were run on Stata/MP, version 18 (see above) and JASP 0.19.3 (University of Amsterdam, Amsterdam, The Netherlands) (54).

## RESULTS

### Study sample characteristics

Table 1 shows that the participants were mostly middle-aged (35–55 years). About two thirds were men, almost all of Bulgarian ethnicity, and half had higher education and lived in Plovdiv. Income adequacy was generally perceived as suboptimal. All participants were confirmed obese according to their BMI, which ranged from 30 to 88. Most residences were in areas with moderate-to-high levels of air pollution and noise and were surrounded by relatively low tree cover and moderate greenness. About half of the participants spent less than 2 h/week in nature.

**Table 1:** Characteristics of the study participants

**Characteristics**	**N**	**Descriptive statistics**		
		**Total sample**	**Plovdiv**	**Other settlements**
***Sociodemographics***				
Age [years] (mean±SD)	90	45.64±6.67	46.48±6.07	44.77±7.21
Male sex (N, %)	90	58 (64.44)	28 (60.90)	30 (68.20)
Bulgarian ethnicity (N, %)	90	83 (92.22)	41 (89.10)	42 (95.50)
Higher education (N, %)	88	44 (48.89)	21 (47.70)	23 (50.00)
Perceived income [1–6 Likert scale] (median; IQR)	65	2.00 (1.00)	3.00 (1.00)	2.00 (1.00)
***Cytokines***				
IL-6 [pg/mL] (median; IQR)	90	28.50 (35.18)	23.10 (42.82)	29.50 (32.52)
TNF-α [pg/mL] (median; IQR)	90	9.80 (19.30)	8.70 (16.90)	12.07 (20.01)
CRP [mg/L] (median; IQR)	88	9.00 (14.00)	5.50 (14.00)	10.50 (12.75)
Visfatin [ng/mL] (median; IQR)	90	6.30 (4.03)	6.30 (3.90)	6.30 (4.08)
Leptin [ng/mL] (median; IQR)	90	46.50 (60.58)	40.3 (53.32)	59.40 (66.88)
Adiponectin [μg/mL] (median; IQR)	90	7.62 (5.01)	8.25 (5.58)	6.58 (4.39)
Adiponectin-leptin ratio (median; IQR)	90	0.13 (0.22)	0.14 (0.22)	0.13 (0.17)
***Adiposity-related markers***				
BMI [kg/m^2^] (mean±SD)	90	39.65±6.50	39.37 (6.34)	39.96 (6.74)
WHR (mean±SD)	90	1.10±0.10	1.09 (0.10)	1.10 (0.10)
Body fat [%] (mean±SD)	90	38.56±7.91	39.32 (7.91)	37.77 (7.92)
Visceral fat [%] (median; IQR)	90	20.00 (4.00)	17.85 (3.27)	17.52 (3.08)
***Environmental factors***				
NO_2_ [μg/m^3^] (median; IQR)	83	22.49 (11.53)	27.04 (5.09)	15.38 (2.83)
PM_2.5_ [μg/m^3^] (median; IQR)	83	21.66 (5.57)	22.09 (2.17)	17.94 (7.44)
PM_10_ [μg/m^3^] (median; IQR)	83	33.25 (7.41)	37.08 (4.72)	30.17 (6.52)
Solid fuel burning (N, %)	90	37 (41.11)	2 (4.35)	35 (79.55)
L_den_ [dB] (median; IQR)	42	68.61 (2.82)	68.61 (2.82)	-
NDVI (median; IQR)	84	0.39 (0.09)	0.39 (0.09)	0.39 (0.10)
Tree cover [%] (median; IQR)	83	4.77 (7.15)	7.87 (6.23)	2.29 (3.31)
Time in nature [hours/week] (median; IQR)	90	2.00 (2.50)	2.00 (2.87)	3.00 (2.13)
***Other covariates***				
Smoker (N, %)	90	52 (57.78)	26 (56.52)	26 (59.09)
Self-rated health [1–5 Likert scale] (median; IQR)	88	2.00 (1.00)	2.00 (1.00)	3.00 (1.00)
Plovdiv city (N, %)	90	46 (51.11)	-	-
Population [people] (median; IQR)	42	3402.50 (1487)	3402.50 (1487)	-
Building type (N, %)	90			
Apartment building		43 (47.78)	35 (76.09)	8 (18.18)
Detached house		17 (18.89)	3 (6.52)	14 (31.82)
Multifamily house		28 (31.11)	8 (17.39)	20 (45.45)
Other		2 (2.22)	0 (0)	2 (4.55)

BMI – body mass index; CRP – C-reactive protein; IQR – interquartile range; IL-6 – interleukin-6; L_den_ – A-weighted day-evening-night sound levels; NDVI – normalized difference vegetation index; NO_2_ – nitrogen dioxide; PM_10_ – particulate matter with aerodynamic diameter ≤10 μm; PM_2.5_ – particulate matter with aerodynamic diameter ≤2.5 μm; SD – standard deviation; TNF-α – tumour necrosis factor alpha; WHR – waist-to-hip ratio

Compared to participants from other settlements, those living in Plovdiv had lower levels of inflammatory markers. They were also exposed to higher levels of air pollution, except for solid fuel burning, which was less prevalent in Plovdiv. Additionally, they lived in areas with higher tree cover density.

### Bivariate correlations

**Figure 1:**
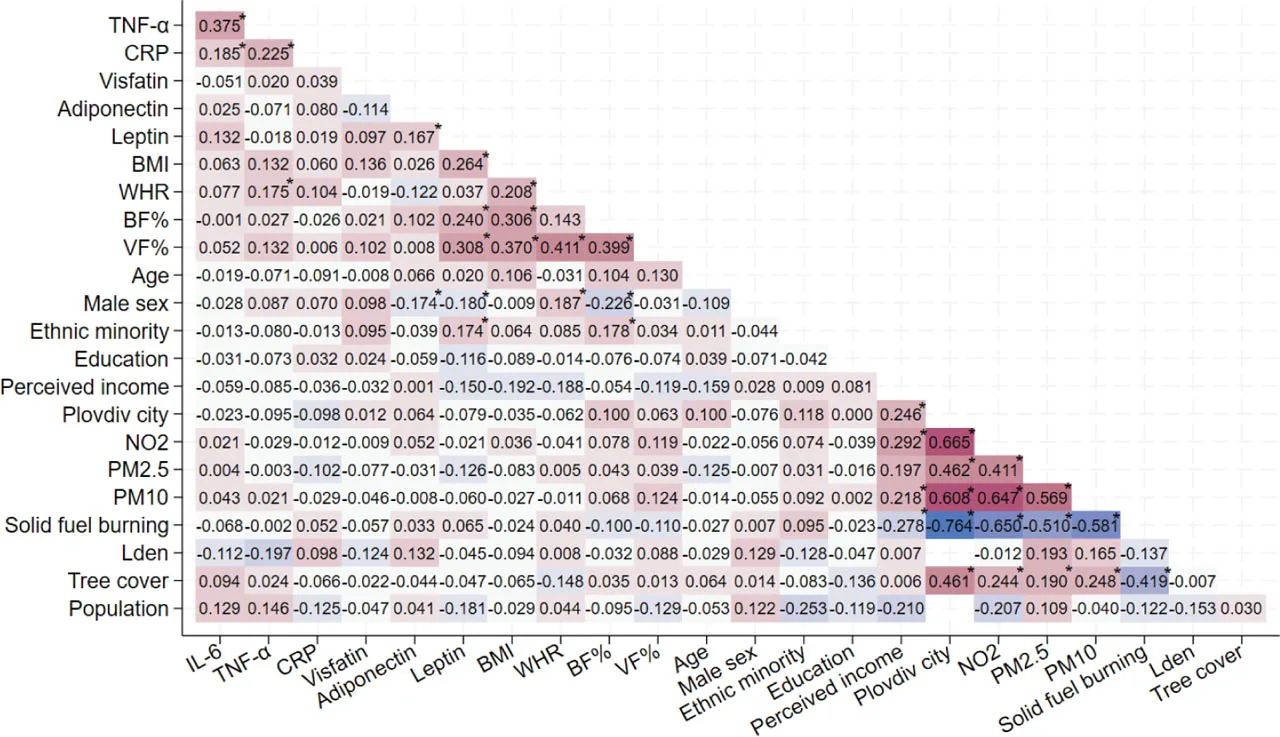
Kendall's tau correlations between proinflammatory markers, adipokines, environmental factors, and other covariates (warmer red shades represent higher positive and colder blue shades represent higher negative correlations) * p<0.05. BF% – body fat percent; BMI – body mass index; CRP – C-reactive protein; IL-6 – interleukin-6; L_den_ – A-weighted day-evening-night sound levels; NDVI – normalized difference vegetation index; NO_2_ – nitrogen dioxide; PM_10_ – particulate matter with aerodynamic diameter ≤10 μm; PM_2.5_ – particulate matter with aerodynamic diameter ≤2.5 μm.TNF-α – tumour necrosis factor alpha; VF% – visceral fat percent; WHR – waist-to-hip ratio

Figure 1 shows that proinflammatory cytokines and adiposity markers significantly correlate with residential exposure parameters besides the expected mutual positive correlations (e.g. TNF-α with WHR and leptin with BMI, BF%, VF%). NO_2_ and particulate air pollutants also positively correlate, as do NDVI and tree cover. Air pollutants have a significant negative correlation with solid fuel burning and NDVI but a positive correlation with tree cover. NDVI positively correlates with population density. Plovdiv residents reporting better income were exposed to higher air pollution, but fewer burned solid fuel at home. Partial correlations controlling for age and sex did not change the observed patterns (data not shown).

### Multivariate models

**Figure 2:**
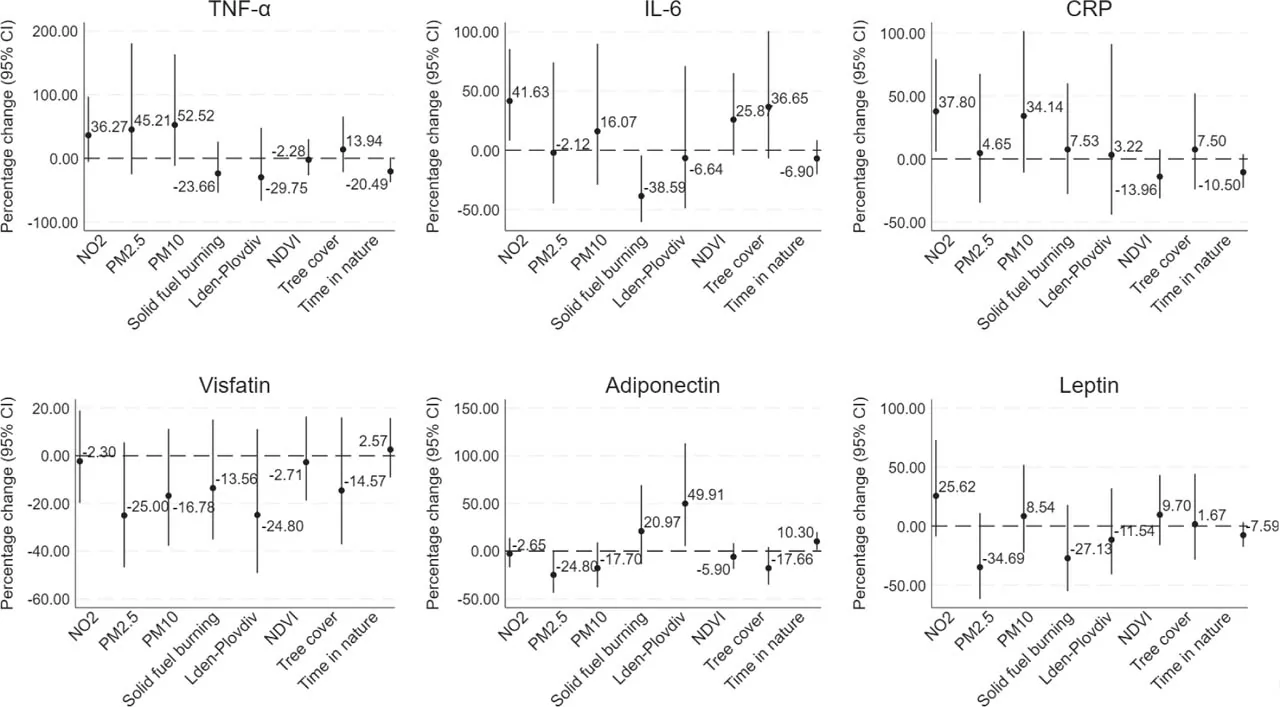
Percentage change in cytokine levels in association with environmental factors. The models are adjusted for age, sex, ethnicity, education, perceived income adequacy, and settlement (for the noise model, settlement was replaced by population). The exposures are tested one-at-a-time in separate models. Coefficients shown are percentage change scores with 95 % confidence intervals, expressed per 10 μg/m^3^ NO_2_, PM_2.5_, and PM_10_, per 5 dB L_den_, per 0.1 in NDVI, per 10 % tree cover, and per 2 h/week in nature. Confidence intervals not crossing the horizontal reference line indicate statistically significant estimates. CRP – C-reactive protein; IL-6 – interleukin-6; L_den_ – A-weighted day-evening-night sound levels; NDVI – normalised difference vegetation index; NO_2_ – nitrogen dioxide; PM_10_ – particulate matter with aerodynamic diameter ≤10 μm; PM_2.5_ – particulate matter with aerodynamic diameter ≤2.5 μm; TNF-α – tumour necrosis factor alpha

**Figure 3:**
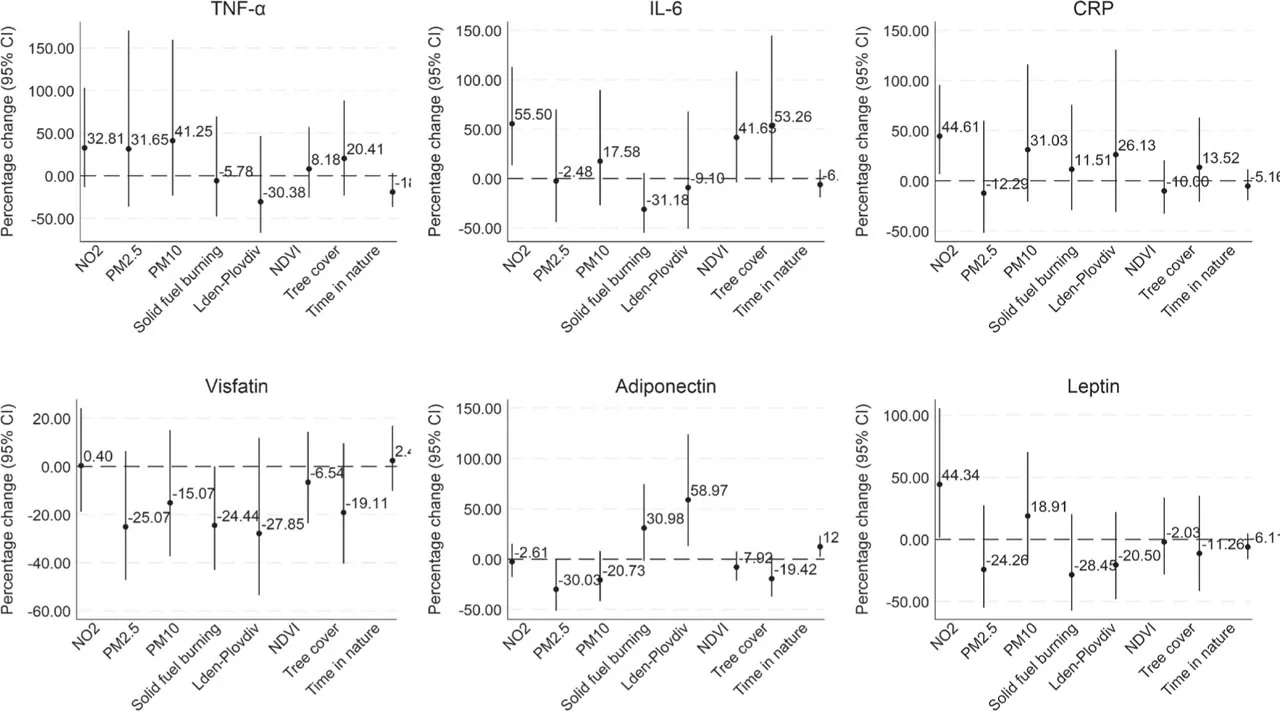
Percentage change in cytokine levels in association with environmental exposures – fully adjusted models. The models are adjusted for age, sex, ethnicity, education, perceived income adequacy, self-rated health, building type, smoking, and settlement (for the noise model, settlement was replaced by population). The exposures are tested one-at-a-time in separate models. Coefficients shown are percentage change scores with 95 % confidence intervals, expressed per 10 μg/m^3^ NO_2_, PM_2.5_, and PM_10_, per 5 dB L_den_, per 0.1 in NDVI, per 10 % tree cover, and per 2 h/week in nature. Confidence intervals not crossing the horizontal reference line indicate statistically significant estimates. CRP – C-reactive protein; IL-6 – interleukin-6; L_den_ – A-weighted day-evening-night sound levels; NDVI – normalised difference vegetation index; NO_2_ – nitrogen dioxide; PM_10_ – particulate matter with aerodynamic diameter ≤10 μm; PM_2.5_ – particulate matter with aerodynamic diameter ≤2.5 μm; TNF-α – tumour necrosis factor alpha

Figure 2 presents percentage change estimates for the cytokines per unit increase in exposure variables. For every 10 μg/m^3^ increase in NO_2_, IL-6 and CRP levels are 41.63 % (95 % CI: 8.16 %, 85.46 %) and 37.80 % (95 % CI: 5.89 %, 79.32 %) higher, respectively. The associations with TNF-α (36.27 %; 95 % CI: −5.66 %, 96.83 %) and leptin (25.61 %; 95 % CI: −8.73 %, 72.88 %) are less precise but have the same direction. A similar direction is observed for PM_2.5_ and PM_10_ and TNF-α and PM_10_ and CRP. Every 2 h/week spent in nature is associated with lower TNF-α (−20.49 %; 95 % CI: −37.56 %, 1.23 %) and higher adiponectin (10.30 %; 95 % CI: 1.36 %, 20.03 %). Associations between time in nature and IL-6, CRP, and leptin also show this direction but are less precise. However, higher NDVI, tree cover, and L_den_ are either negatively associated with cytokines or not at all. These patterns persist in the fully adjusted models (Figure 3), although the effect estimates for NO_2_ and time in nature are somewhat higher.

**Figure 4:**
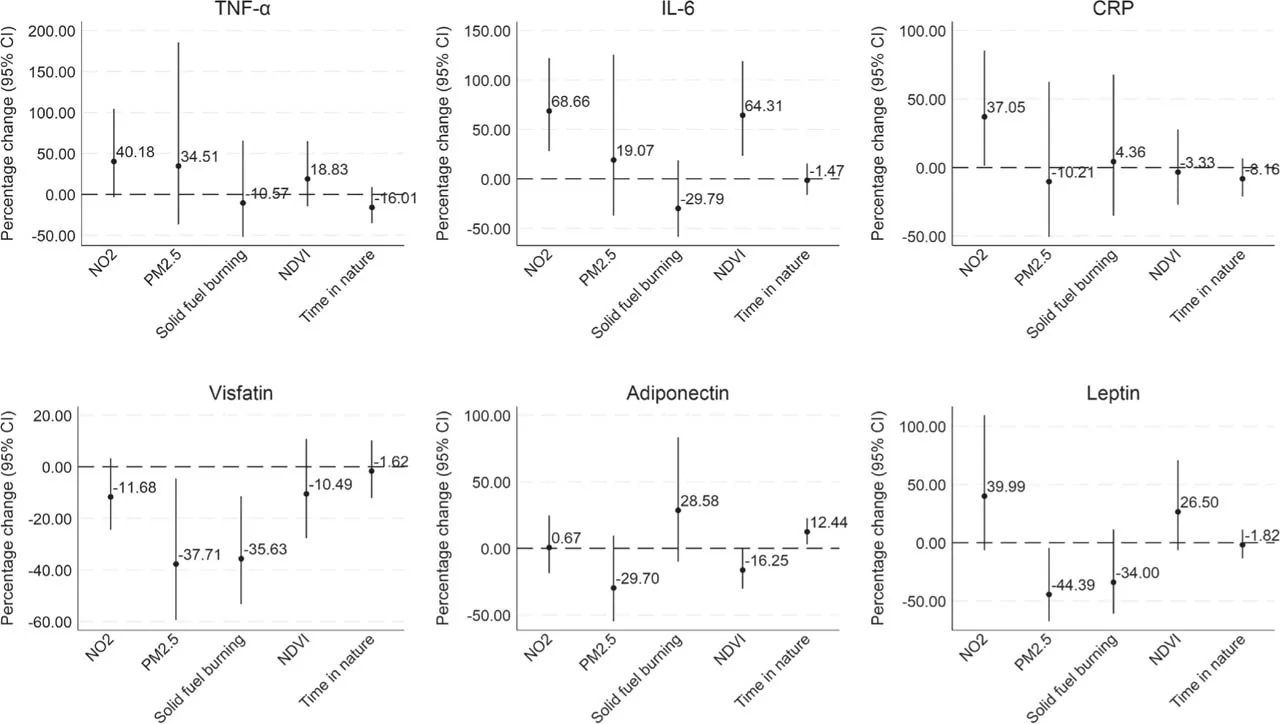
Percentage change in cytokine levels in association with environmental exposures – multi-exposure models. The models are adjusted for age, sex, ethnicity, education, perceived income adequacy, and settlement (for the noise model, settlement was replaced by population). The exposures are adjusted for each other and tested all at once. Coefficients shown are percentage change scores with 95 % confidence intervals, expressed per 10 μg/m^3^ NO_2_, PM_2.5_, per 0.1 in NDVI, and per 2 h/week in nature. Confidence intervals not crossing the horizontal reference line indicate statistically significant estimates. CRP – C-reactive protein; IL-6 – interleukin-6; NDVI – normalised difference vegetation index; NO_2_ – nitrogen dioxide; PM_2.5_ – particulate matter with aerodynamic diameter ≤2.5 μm; TNF-α – tumour necrosis factor alpha

In multi-exposure models, the results for IL-6 and time spent in nature remain consistent. Meanwhile, NDVI is associated with higher levels of IL-6 and lower levels of adiponectin. Additionally, PM_2.5_ and solid fuel burning are associated with lower levels of leptin and visfatin (Figure 4). Additional adjustment of the associations between environmental factors and inflammatory markers for visceral fat has not materially changed the effect sizes or precision (data not shown).

**Figure 5:**
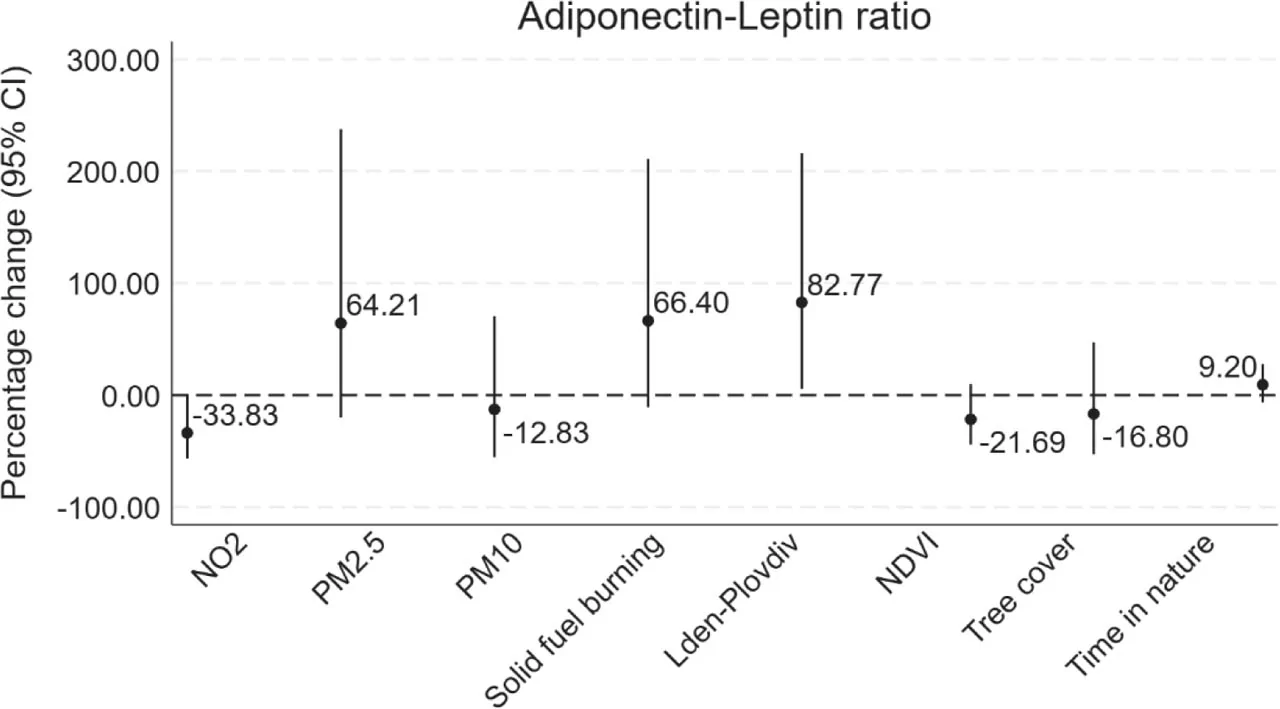
Percentage change in adiponectin-leptin ratio in association with environmental factors. The models are adjusted for age, sex, ethnicity, education, perceived income adequacy, and settlement (for the noise model, settlement was replaced by population). The exposures are tested one-at-a-time in separate models. Coefficients shown are percentage change scores with 95 % confidence inter vals, expressed per 10 μg/m^3^ NO_2_, PM_2.5_, and PM_10_, per 5 dB L_den_, per 0.1 in NDVI, per 10 % tree cover, and per 2 h/week in nature. Confidence intervals not crossing the horizontal reference line indicate statistically significant estimates. L_den_ – A-weighted day-evening-night sound levels; NDVI – normalised difference vegetation index; NO_2_ – nitrogen dioxide; PM_10_ – particulate matter with aerodynamic diameter ≤10 μm; PM_2.5_ – particulate matter with aerodynamic diameter ≤2.5 μm

The main model for the adiponectin-leptin ratio reveals a negative association with NO_2_ (-33.83 %; 95 % CI: −56.59 %, 0.85 %), while the associations with the other exposures are imprecise and unexpected, such as the positive association with solid fuel burning and L_den_ and the negative one with NDVI (Figure 5).

**Figure 6:**
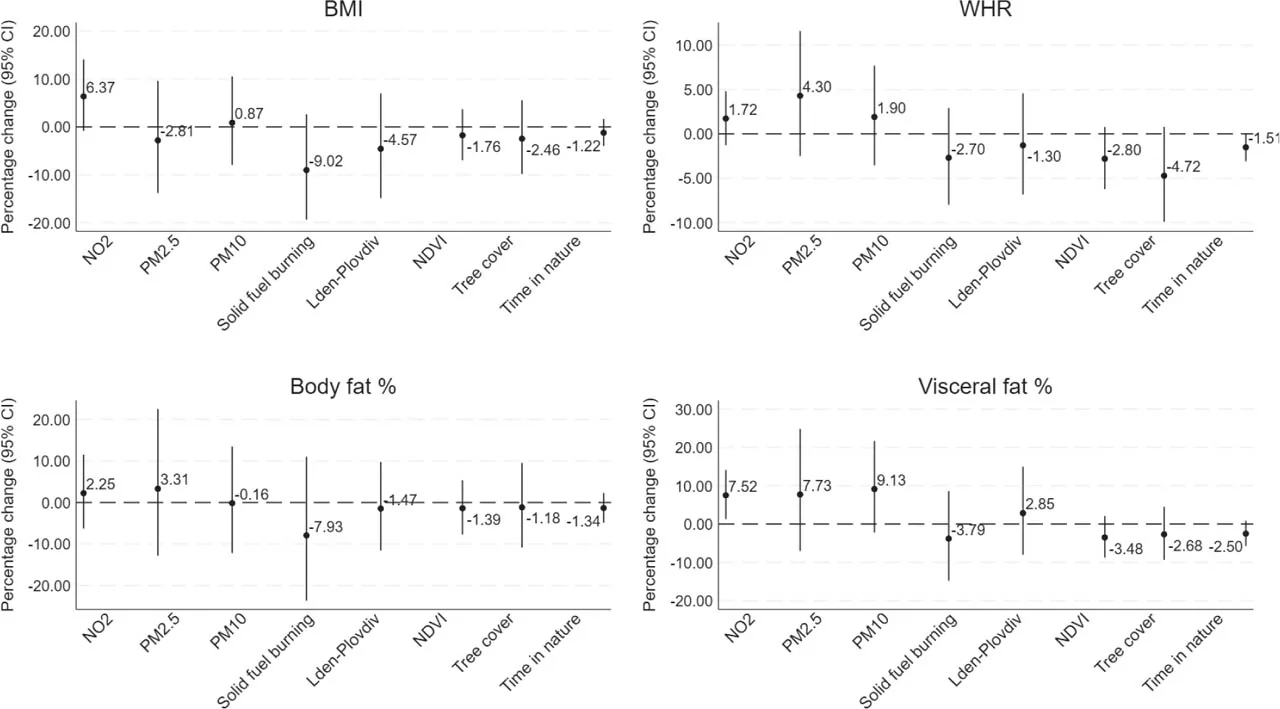
Percentage change in adiposity markers in association with environmental factors. The models are adjusted for age, sex, ethnicity, education, perceived income adequacy, and settlement (for the noise model, settlement was replaced by population). The exposures are tested one-at-a-time in separate models. Coefficients shown are percentage change scores with 95 % confidence intervals, expressed per 10 μg/m^3^ NO_2_, PM_2.5_, and PM_10_, per 5 dB L_den_, per 0.1 units in NDVI, per 10 % tree cover, and per 2 h/week in nature. Confidence intervals not crossing the horizontal reference line indicate statistically significant estimates. BMI – body mass index; L_den_ – A-weighted day-evening-night sound levels; NDVI – normalised difference vegetation index; NO_2_ – nitrogen dioxide; PM_10_ – particulate matter with aerodynamic diameter ≤10 μm; PM_2.5_ – particulate matter with aerodynamic diameter ≤2.5 μm; WHR – waist-to-hip ratio

Higher NO_2_ is associated with higher BMI (6.37 %; 95 % C: −0.82 %, 14.08 %) and VF% (7.52 %; 95 % C: 1.30 %, 14.12 %) (Figure 6). Although the confidence intervals are wider, PM_2.5_ and PM_10_ are also positively associated with WHR and VF%. NDVI, tree cover, and time spent in nature are associated with lower WHR. Further adjustments have not affected NO_2_ associations, but the one for time spent in nature has dropped considerably. Adjusting for other exposures has not changed these results, but has for NDVI, tree cover, and solid fuel burning associations with lower WHR.

In the subgroup of participants residing in Plovdiv, the effect estimates for NO_2_ are lower but remain consistent in their direction. More time in nature is associated with higher adiponectin and lower WHR. The unlikely associations for NDVI, tree cover, L_den_, PM_2.5_, and PM_10_, especially prominent with visfatin, have persisted after adjustment.

## DISCUSSION

### Overall findings and interpretation

We found an association between higher long-term exposure to NO_2_ and higher inflammatory and adiposity markers in our study sample of obese adults with MetS. Conversely, greater time spent in nature is inversely associated with certain inflammatory and adiposity markers.

Regarding the other environmental factors, we observed null, inconsistent, or occasionally unexpected protective association, for example, between the adiponectin-leptin ratio and solid fuel burning or higher L_den_, and lower NDVI. These findings have remained largely consistent after adjustment for sociodemographic, area, and general health covariates, and most have persisted in multi-exposure models.

Although there is a consensus that a key pathway behind the detrimental effects of air pollution and traffic noise is the induction of chronic low-grade inflammation, the observational evidence in humans is heterogeneous, even for air pollution, which has received much more attention than noise and greenspace. A meta-analysis of 38 studies confirms the effects of air pollution on inflammatory biomarkers but also concludes that they are stronger short- than long-term (12). Another meta-analysis (13) reports increases of 18.01 % and 5.61 % in CRP levels for every 10 μg/m^3^ increment in ambient PM_2.5_ and PM_10_. Contrary to these findings, our study has revealed no association of inflammation with particulate matter but did the one with NO_2_. This finding aligns with a Chinese study (55) reporting an increase in serum CRP levels with an increment in NO_2_, which was also observed by Liu et al. (56). However, contrasting results were reported by the German KORA study (57), where particulate matter was marginally associated with elevated CRP and IL-6 levels. Our divergent findings may be owed to different exposure data. Although our PM_2.5_ LUR model demonstrated a higher R^2^ than the NO_2_ model, given the model's development in a pan-European context, the input data quality may have been suboptimal for accurately estimating PM_2.5_ in Bulgaria. Very few air quality monitoring stations in the country measure PM_2.5_ (only one in Plovdiv), and one study (58) reported NO_2_ to be more consistently associated with hospital admissions for cardiometabolic and respiratory diseases than either PM_2.5_ or PM_10_.

Furthermore, our results do not support that domestic burning of solid fuel would be associated with harmful outcomes. A similar finding of no harmful effect was reported among Chinese adults (59). However, the lack of information on the type of solid fuel participants used, use behaviours, and personal exposure could have contributed to the lack of the expected association between solid fuel use in the home and the studied biomarkers.

Most research on the effects of air pollution on other cytokines, such as TNF-α, has focused on short-term exposures, particularly in relation to particulate matter (12, 60, 61). Moreover, the knowledge regarding the effects of gaseous air pollutants on adipokines secreted by the adipose tissue is limited. Leptin may increase following PM exposure (62, 63) and, in turn, upregulate secretion of TNF-α and IL-6 (16), while adiponectin levels may be lowered by PM (57, 63). However, other exposure-biomarker relationships are poorly investigated, and we are unaware of another study that has explored associations between long-term residential exposures and visfatin. While we found no clear associations, investigating such alternative biomarkers may shed more light on how the environment can shape metabolic inflammation.

As for noise, our study could not corroborate experimental findings that it contributes to inflammation (21). Only a few epidemiological studies have assessed chronic traffic noise exposure and the biomarkers studied here. A study based on the UN Biobank data did not find robust evidence of elevated CRP levels with higher L_den_ levels (64). A suggestive trend was observed in another study (65), which demonstrated a non-significant positive association between L_den_ exceeding 70 dB and CRP. In our study, the relationship between L_den_ and CRP is non-linear and plateaus above 67.5 dB. It remains imprecise in both linear and non-linear models, possibly because noise data were available for only half of the sample, for participants who resided in Plovdiv.

The observed associations of NO_2_ with cytokines and the adiponectin-leptin ratio of <1 in our study suggest that populations with metabolic dysfunction may be strongly affected by proinflammatory exposures. One study (15) reported significant increases in leptin and CRP with higher NO_2_ and PM in prediabetic adults than in healthy controls (15), while another (66) reports higher leptin levels in serum and adipose tissue of obese young adults living near a road.

However, our findings also suggest that obese individuals may benefit from some exposures, such as spending time in nature, even though this protective association is somewhat diminished in multi-exposure models. Some studies report that forest bathing and waterfall exposure therapies reduce proinflammatory cytokine levels (24, 25, 67) and one systematic review (68) suggests protective effects of nature-based therapies against inflammation. Recent research exploring the anti-inflammatory effects of frequent residential greenspace exposure reports lower inflammation scores and lower levels of IL-6 and CRP (26–28). Furthermore, the presence of greenspace in residential areas has been reported to inversely correlate with leptin levels (69). These findings – that exposure to nature promotes a healthier immune response (26) and weight status (70) – have been corroborated by ours. The mechanisms that may explain these effects include stress reduction, microbiome diversity, increased physical activity, and social engagement (29). Interestingly, however, we have found that greater NDVI and tree cover are associated with adverse outcomes, suggesting a potentially harmful effect. The selection of tree cover as the indicator of greenspace was based on the belief that trees provide significant stress-reducing and pollution-mitigating benefits (71). However, a closer inspection of the spatial distribution of tree cover in our study areas revealed a higher density of trees around the homes of participants in Plovdiv than in other settlements. Therefore, it can be argued that this variable may have inadvertently served as a proxy for urbanicity, reflecting the harm contributed by other spatially correlated exposures, such as air pollution. Another aspect can be urban morphology types and the level of utilisation of urban greenspace. For instance, multifamily apartment modernist buildings in large housing estates were developed in the 1950s to 1980s utilising abundant open space, with surroundings often well covered by trees. In future research, alternative greenspace types should be investigated to ascertain whether they exert different effects.

### Implications

Our results may inform future investigations of inflammatory cytokines and adipokines that have been previously unexplored and have unique, frequently synergistic, systemic effects (72). Formal tests of the causal pathways between inflammatory and metabolic biomarkers through mediation analysis are needed to partial out the indirect effects of environmental factors on these outcomes. In terms of chronic diseases, identifying detrimental and potentially beneficial drivers is paramount, particularly given the preponderance of cardiometabolic disorders as the primary cause of premature mortality worldwide (73). A comprehensive understanding of the environmental determinants of metabolic inflammation can guide the development of effective preventive strategies that go beyond classic behavioural and biomedical risk factors. Research on the effects of air pollution, for instance, has prompted interest in individual-level coping strategies, such as behaviour modification, which can mitigate exposure to air pollution or even offset the inflammatory and oxidative pathways activated by air pollution (74). A diet rich in antioxidants and anti-inflammatory substances, such as omega-3 fatty acids, can reduce oxidative stress and inflammation levels (75) and can blunt adverse response to air pollution (76,77,78) and reduce cardiovascular risk (79). Therefore, more mechanistic findings are required to justify interventions that not only aim to reduce exposure levels but also enhance individual biological resilience by counteracting inflammation.

Our findings are consistent with the evidence that engagement with natural environments may support the immune system functioning and weight control through various nature-based therapies (80), some of which rely on the benefits of stress reduction, physical activity, natural aerosols, and soundscapes. As posited by White et al. (30), the biopsychosocial resilience theory exemplifies the various levels at which nature contact could support health, not only by primary prevention, but also in vulnerable populations at risk, such as obese individuals and those with MetS (81, 82).

### Study limitations

This study is not without limitations. The sample size is small, which may have reduced the power of associations, given the number of independent variables in the models. This relatively modest sample size is further reduced to participants from Plovdiv for some of the analyses, and the number of covariates in these models may also have rendered the results biased towards the null and limited the precision of the observed associations.

It is also noteworthy that several confounders are missing a lot of data. We attempted to mitigate this issue by imputing those values, which may have affected the strength of associations. Even so, the associations for NO_2_ and time in nature are consistent across sensitivity analyses.

Another limitation is that the cross-sectional design precludes causal inferences. We included only participants who had resided in the area for at least five years, which means that the exposure variables represent environmental conditions that preceded the outcomes, lending greater biological plausibility to our correlational findings.

Our sample is restricted to obese individuals with MetS. Therefore, it may have had insufficient variability in biomarker levels to allow for a finer observation of exposure-response relationships. For example, the correlations between inflammation and adiposity markers and between smoking and biomarkers are likely underestimated in the absence of normal-weight individuals in the sample.

Another limitation is that we used only the available data on certain exposures, which, for instance, precluded the modelling of traffic noise effects for participants residing outside of Plovdiv. The relatively high levels of road traffic noise in Plovdiv and the limited spatial contrast they offer (83) could have prevented us from observing L_den_ associations in the present study. There were also some systematic differences in environmental factors and inflammation markers between Plovdiv and other settlements. As in similar studies, we lacked personal exposure data that would capture microenvironments and people's daily activities.

Another caveat regarding exposure assessment concerns the implausible associations with many of the inflammatory outcomes, including solid fuel burning, L_den_, and GIS-derived greenness. We speculate that this is due to the specific patterns of associations between the exposures, which prevented us from disentangling protective from harmful effects. However, this is only a partial explanation, and we suggest caution in interpreting these results. Furthermore, due to a temporal mismatch in the years for which objective exposure data were available, we had to assume that spatial relationships remained the same, and so did the correlations across the years (84). We expect the resulting bias to be low when exposure variables are analysed as continuous independent variables. However, since some of the measured biomarkers (e.g. interleukins) vary considerably over time, it would have been preferable to have had exposure data for a matched period.

Despite the implementation of rigorous exclusion criteria, which effectively mitigated the influence of numerous comorbidities, the impact of specific medications was not fully addressed. This omission could have led to an underestimation of certain associations. For instance, the use of statins has been speculated to obscure the effect of air pollution on CRP (31, 85). Additionally, the InBody analyser only approximated body composition estimates for BF% and VF%, but this technology has demonstrated reasonable validity when gold standard methods are unavailable (41). Finally, our findings have limited external validity, as the sample was opportunistic and the participants were recruited at a single site and may not be representative of the obese population with MetS.

## CONCLUSION

Regardless of its limitations, our study identifies an association between long-term exposure to traffic-related air pollution with higher systemic inflammation and adiposity and between time spent in nature and certain inflammation and adiposity markers. Other associations are null, inconsistent, or occasionally unexpected, possibly due to unaccounted confounding factors.

Our findings are not conclusive, though, but set the path for larger longitudinal studies to better understand the environmental determinants of metabolic inflammation, which could, in turn, support the development of alternative preventive strategies.

## Abbreviations

AIC: Akaike information criterion
BIC: Bayesian information criterion
BF%: body fat percent
BMI: body mass index
CRP: C-reactive protein
IL-6: interleukin-6
IQR: interquartile range
L_den_: day-evening-night A-weighted sound levels
LUR: land use regression
NDVI: normalised difference vegetation index
NO_2_: nitrogen dioxide
PM_10_: particulate matter with aerodynamic diameter ≤10 μm
PM_2.5_: particulate matter with aerodynamic diameter ≤2.5 μm
SD: standard deviation
TNF-α: tumour necrosis factor alpha
VF%: visceral fat percent
WHR: waist-to-hip ratio
